# Fellow-Workers and the Roll of Honour

**Published:** 1915-05-01

**Authors:** 


### ROUND THE WORLD.

[The Editor invites Early Information and Contributions Marked "Fellow-Workers."]

### Fellow-Workers and the Roll of Honour.

A compliment has been paid to the work of the Australian doctors by the grant of the rank of honorary surgeon-general in the British Army to Surgeon-General William Daniel Campbell Williams, C.B., who has been directing the Commonwealth military medical service. Surgeon-General Williams studied medicine at University College Hospital, qualifying M.R.C.S. Eng. in 1879, and, taking up his active life in the Colonies, had a successful career in Sydney, where he became attached to the St. Vincent's Hospital, and climbed his way to the responsible position of senior surgeon there. On retirement he was given his present appointment of consulting surgeon to the institution.

Another distinguished Australian surgeon who has been working on behalf of the wounded is Mr. Robert Hamilton Russell, of Melbourne, who was for a time at the Australian Voluntary Hospital, Wimereux. A King's College Hospital man he qualified in 1882, and a few years later became an F.R.C.S. Eng. In Melbourne, whither he has just returned, Mr. Russell has built up for himself an honoured position, his public appointments including those of senior surgeon to the Alfred Hospital and consulting surgeon to three other important institutions there —the Children's Hospital, the Eye and Ear Hospital, and the Victoria Hospital for Women. A variety of contributions to surgical literature have come from his pen.

Dr. William Hunter, who is with the gallant band of medical men in Serbia, is not only one of our chief authorities on fevers, but has a world-wide reputation for his work in connection with diseases of the blood. Much of present-day knowledge of malignant types of anæmia we owe to his researches. A keen supporter of Charing Cross Hospital, where he holds the post of senior assistant physician and lecturer on pathology, Dr. Hunter is a brilliant son of the Edinburgh school. Qualifying in 1883, and subsequently taking a gold medal at the M.D. Edin., he speedily distinguished himself on settling in London, and in due course was elected to the fellowship of the Royal College of Physicians. An examiner in medicine for Glasgow University, he is also physician to the London Fever Hospital.

The late Captain Maurice Burnett, R.A.M.C., killed in the Persian Gulf campaign, joined the Army just over three years ago, after serving as senior house physician at St. George's Hospital. A promising student, he qualified L.R.C.P., M.R.C.S. in 1911. His father, who is alive to mourn this loss, had a distinguished military career, retiring with the rank of surgeon-general.

A genuinely keen officer has been lost to the Army by the death of Lieutenant Ernest Stratford, R.A.M.C., who was seriously wounded in the fighting round Neuve Chapelle on March 17. Although not killed outright, the shock and subsequent infection proved too much for his strength, and he died in this country about a month afterwards. Lieutenant Stratford, although only thirty-nine years of age, had packed a great variety of experiences into his life. Educated at Cambridge, he saw military service long before he became a medical man, serving in South Africa with Bethune's Mounted Infantry, when he attained captain's rank. Subsequently he pursued his medical work at St. Mary's, qualifying L.R.C.P., M.R.C.S., and afterwards holding a resident appointment at the West End Hospital for Nervous Diseases. Amongst original work he recorded observations on the blood-pressure during aeroplane flights, and made practical deductions therefrom. Last year he joined the R.A.M.C., and worked at Netley and the Bournemouth Indian Hospital before being sent to the Front.

Dr. Isabel Ormiston, who is proceeding to Montenegro under the auspices of the Wounded Allies' Relief Committee, graduated at Sydney University. For several years she has been Chief Medical Inspector of Schools in Tasmania, and has specialised in the development of disease-prevention methods.

The late member for North Tipperary, Captain John Esmonde, joined the R.A.M.C. last autumn, and was allotted duties in his native country with the rank of captain. A diplomate of the Irish Royal College of Surgeons, he qualified in 1884, and after practising for many years in England he went back to Ireland, and, engaging in politics, was returned for the Nationalist Party five years ago. A man of little over fifty years of age, his early death from pneumonia is much lamented by a wide circle.

The names of several eminent civilians and R.A.M.C. officers appear in connection with the proposal to compile a systematic history of the war in its medical and surgical bearings. As at present announced, the organising committee will consist of Sir William Osler, M.D., F.R.S., and Lieutenant-Colonel O. L. Robinson, R.A.M.C., representing medicine; Colonel Sir William Leishman, C.B., F.R.C.P., F.R.S., and Captain F. W. Andrewes, for pathology and bacteriology; Colonel F. F. Burghard, M.D., M.S., F.R.C.S., and Lieutenant-Colonel E. M. Pilcher, D.S.O., M.B., F.R.C.S., R.A.M.C., to supervise the section of surgery; Colonel W. H. Horrocks, M.B., K.H.S., and Lieutenant-Colonel W. W. O. Beveridge, D.S.O., M.B., R.A.M.C., for hygiene and sanitation; with Lieutenant-Colonel H. P. W. Barrow, R.A.M.C., Dr. John Brownlee, M.D., D.Sc., and Lieutenant-Colonel W. N. Barrow, M.V.O., R.A.M.C., for statistics.

This learned committee will meet under the presidency of the Director-General of the Army Medical Service, Sir Arthur Thomas Sloggett, K.C.B., C.M.G.; whilst Dr. W. M. Fletcher, M.D., Sc.D., F.R.S., and Captain F. S. Brereton will superintend the secretarial work and the classification of documents.

Captain Archibald Alfred Sutcliff, R.A.M.C., a prisoner in Germany, has lately succumbed to typhus fever. First missed after the action at Mons in August, it ultimately transpired that he had been captured with several colleagues. Recently he had been at Wittenburg, where he contracted the deadly infection. A St. Thomas's man, Captain Sutcliff qualified ten years ago, taking both the British conjoint diploma and the double degree (M.B., B.S.) of London University. In the following year he entered the R.A.M.C. His death at the early age of thirty-four years, occurring after he had escaped the perils of the battlefield, is much to be deplored.

